# Primaquine ineligibility in anti-relapse therapy of *Plasmodium vivax* malaria: the problem of G6PD deficiency and cytochrome P-450 2D6 polymorphisms

**DOI:** 10.1186/s12936-018-2190-z

**Published:** 2018-01-22

**Authors:** J. Kevin Baird, Katherine E. Battle, Rosalind E. Howes

**Affiliations:** 1 Eijkman-Oxford Clinical Research Unit, Jalan Diponegoro No.69, Central Jakarta, 10430 Indonesia; 2 Centre for Tropical Medicine and Global Health, Nuffield Department of Medicine, University of Oxford, Old Road Campus, Oxford, OX3 7FZ UK; 3 Oxford Big Data Institute, Nuffield Department of Medicine, University of Oxford, Old Road Campus, Oxford, OX3 7FZ UK; 4 Center for Global Health and Diseases, Case Western Reserve University, Biomedical Research Building, 2109 Adelbert Road, Cleveland, OH 44106-4983 USA

**Keywords:** *Plasmodium vivax*, Primaquine therapy, Contraindications for anti-relapse therapy, G6PD deficiency, CYP2D6

## Abstract

The hypnozoite reservoir of *Plasmodium vivax* represents both the greatest obstacle and opportunity for ultimately eradicating this species. It is silent and cannot be diagnosed until it awakens and provokes a clinical attack with attendant morbidity, risk of mortality, and opportunities for onward transmission. The only licensed drug that kills hypnozoites is primaquine, which attacks the hypnozoite reservoir but imposes serious obstacles in doing so—at hypnozoitocidal doses, it invariably causes a threatening acute haemolytic anaemia in patients having an inborn deficiency in glucose-6-phosphate dehydrogenase (G6PD), affecting about 8% of people living in malaria endemic nations. That problem excludes a large number of people from safe and effective treatment of the latent stage of vivax malaria: the G6PD deficient, pregnant or lactating women, and young infants. These groups were estimated to comprise 14.3% of populations resident in the 95 countries with endemic vivax malaria. Another important obstacle regarding primaquine in the business of killing hypnozoites is its apparent metabolism to an active metabolite exclusively via cytochrome P-450 isozyme 2D6 (CYP2D6). Natural polymorphisms of this allele create genotypes expressing impaired enzymes that occur in over 20% of people living in Southeast Asia, where more than half of *P. vivax* infections occur globally. Taken together, the estimated frequencies of these primaquine ineligibles due to G6PD toxicity or impaired CYP2D6 activity composed over 35% of the populations at risk of vivax malaria. Much more detailed work is needed to refine these estimates, derive probabilities of error for them, and improve their ethnographic granularity in order to inform control and elimination strategy and tactics.

## Background

The 6th International Conference for *Plasmodium vivax* Research at Manaus, Brazil in June of 2017 gathered hundreds of scientists, physicians, and students representing governments, institutions, universities, foundations, companies, and societies engaged on that topic. The expanding discovery of crucial gaps in understanding this infection, and the will to close them, explains the growth and vigor of this conference series since the first held in Bangkok 15 years ago.

Research on *P. vivax* languished in the latter 20th century under a false aegis of harmlessness. The dogma of a benign species, rooted in pre-Laveran taxonomies of disease, today yields to evidence of a pernicious infection capable of great harm. The realization of that harm being done, and the multiple opportunities for rapidly and effectively doing something about it, energizes the community of workers on vivax malaria. That energy, almost palpable at Manaus, was stoked by the extraordinary progress being made in bringing knowledge to mitigate this dangerous parasite’s burden. Decades of stymied scientific progress today deeply enrich possibilities for discoveries of great health impacts.

Among the many characteristics of vivax malaria featured in the plenary lectures, posters, and pre-meeting courses, the hypnozoite dominated at Manaus. That stage of *P. vivax* represents at once the toughest challenge and greatest opportunity for progress measured in substantial health dividends quickly achieved. The hypnozoite has been the most neglected aspect of this most neglected species, a seemingly “too hard to do” target of investigation. The prospects for discoveries of great weight, therefore, are relatively very good—if the hypnozoite reservoir can safely and routinely be killed in endemic communities, those populations can almost certainly be freed of the burdens of morbidity and mortality it imposes. Attacking the hypnozoite reservoir is very probably the key to the failure or success of that endeavour, but daunting clinical and technical obstacles now protect it.

## The G6PD deficiency problem

Primaquine is an 8-aminoquinoline drug derived from the first synthetic anti-malarial licensed for clinical use in the 1920s, pamaquine (or plasmochin) [1]. Strategic urgencies of World War II spurred an effort to curb the notorious toxicity of pamaquine, providing primaquine by 1952. That search for 8-aminoquinolines winnowed candidate compounds without knowledge of the basis of its primary toxicity problem—glucose-6-phosphate dehydrogenase (G6PD) deficiency, discovered only in 1956 [2]. Primaquine, like all 8-aminoquinolines, suffers that defect, but it nonetheless became and remains our only means of killing hypnozoites. That flaw threatens the approximately 400 million G6PD-deficient people globally, representing about 8% of people living in malaria endemic countries [3], with a potentially life-threatening acute haemolytic anaemia at therapeutic hypnozoitocidal doses [4]. This problem, and the inability to screen most patients with malaria for G6PD deficiency, accounts for the problematic extended daily dosing of primaquine (14 days), the prudent reluctance to prescribe or demand adherence to it, and the prohibition of its use in pregnant or lactating women and young infants. In these people, the outlines of the challenge in killing the hypnozoite reservoir may be seen—entire groups of substantial numbers of people unable to safely receive primaquine therapy. How many such people are primaquine-ineligible?

## Dimension of the G6PD deficiency problem

**Fig. 1:**
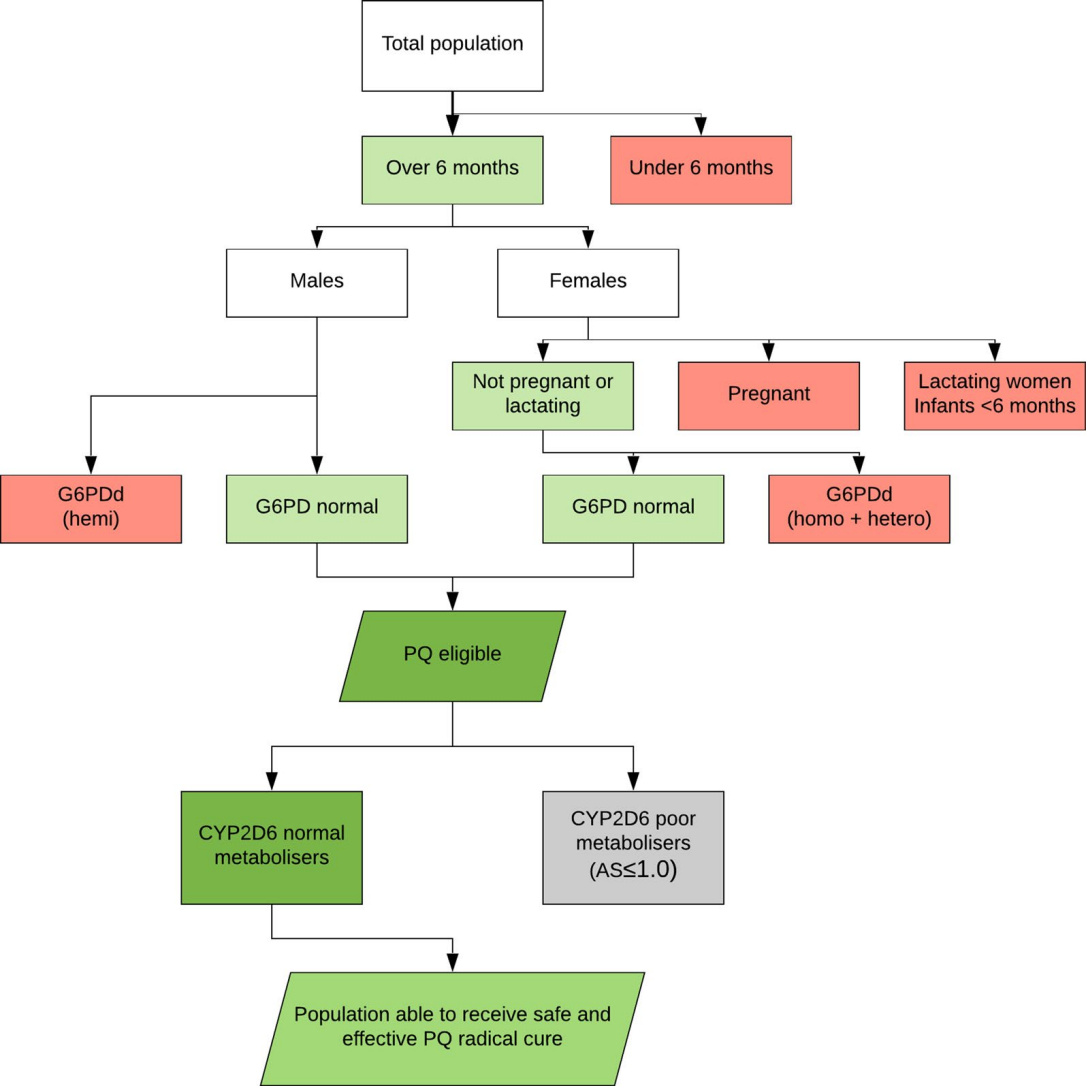
Stepwise exclusions of population subsets ineligible for *Plasmodium vivax* radical cure by primaquine. Red boxes indicate excluded subsets, while green are eligible. *AS* activity score, *G6PDd* G6PD deficient, *hemi* hemizygote, *hetero* heterozygote, *homo* homozygote, *PQ* primaquine

Several available datasets were collated to address this question, as illustrated in Fig. 1. Exclusions of ineligible subsets of the population were applied at the national level and then summed to regional totals. First, infants younger than 6 months were excluded [5]. Second, the proportions of women who at any one point in time are pregnant or breastfeeding infants under 6 months of age were calculated. To estimate numbers of pregnancies per country, age-specific fertility rates [6] were combined with total numbers of females of reproductive age (15–44 years) per 5-year age category, adjusting for rates of abortions [7] and fetal losses, which were based on stillbirth rates [8]. The proportion of lactating women was estimated from breastfeeding rates [9] adjusted to numbers of infants younger than 6 months. And third, the final safety exclusion was G6PD deficiency. National-level estimates of G6PD deficient allele frequencies were used to quantify G6PD deficient subsets of eligible populations [3]. The G6PD gene’s X-linked location means that G6PD deficient allele frequencies correspond exactly to the overall frequencies of deficient males. G6PD deficient females were excluded for being either homozygous or heterozygous carriers, quantified from the population-level allele frequencies following the Hardy–Weinberg model [3]. Altogether, these three step-wise exclusions identified 14.3% of the population across the 95 *P. vivax* endemic countries [10] to be precluded from primaquine radical cure on safety grounds. The majority of those excluded were due to G6PD deficiency (70%), while infants under 6 months and females either pregnant or lactating made up the remaining 12 and 18%, respectively.

## The impaired CYP2D6 problem

Findings described at Manaus point to yet another group of primaquine ineligibles. Studies in Indonesia followed up on the report of Bennett and colleagues [11] suggesting impaired cytochrome P450 2D6 (CYP2D6) function with naturally occurring allelic polymorphisms resulted in therapeutic failure of primaquine against latent *P. vivax* malaria. Impaired CYP2D6 alleles occurred in 95% of therapeutic failures following directly supervised, high-dose primaquine of good quality in subjects followed for a year where reinfection was highly unlikely [12]. Those failures occurred at a rate of 15% among several hundred such treatments (J. Kevin Baird, personal communication, 2017).^1^ Impaired CYP2D6 alleles may commonly occur among people at risk of vivax malaria. The *10 allele, for example, is especially common among the Southeast Asian populations so far surveyed, and more than half of the global burden of *P. vivax* occurs in this region [13]. Much more thorough surveys of these alleles are needed in order to assess the number of people who may metabolize primaquine at levels insufficient for therapeutic success.

## Dimension of the impaired CYP2D6 problem

Available evidence permits preliminary estimation of the proportion of the population in whom primaquine metabolism would likely be insufficient to achieve radical cure against *P. vivax* relapse. The *CYP2D6* gene is highly heterogeneous, with over 150 alleles defined to date [14], and phenotypes ranging from complete dysfunction to ultra-rapid metabolism. Various surveys of the major allelic variants of *CYP2D6* have been conducted among population groups globally which have been compiled by Sistonen et al. [15]. Although variable in terms of the panel of alleles screened, a consistent core of polymorphic variants are usually included in these community cross-sectional surveys. Enzyme activity phenotypes have been determined for individual alleles using proxy indicators of CYP2D6 metabolic activity, and an allele classification system devised by Gaedigk and colleagues categorizes alleles into non-functional, low, normal and increased metabolizers, with respective activity scores of 0.0, 0.5, 1.0 and 2.0 per allele, corresponding to overall phenotype scores of 0.0, 0.5, 1.0, 1.5, 2.0 or > 2.0 (see Table 1) [16]. Individuals with activity scores ≤ 1.0 were considered to be poor primaquine metabolizers, as per the outcomes of the clinical trial by Baird and colleagues (J. Kevin Baird, personal communication, 2017).

**Table 1:**
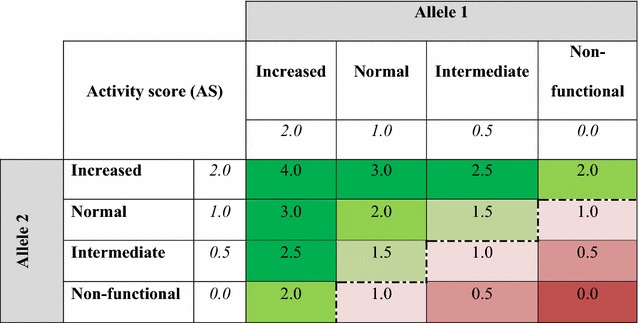
Summary of CYP2D6 phenotype activity score estimations

Activity scores for each component allele in the genotype (allele 1 and allele 2) sum to give the genotype activity scores. Phenotypes with AS ≤ 1.0 (in red) are considered to be poor primaquine metabolizers (J. Kevin Baird, personal communication, 2017). The italic data was to differentiate the allele-level from phenotype-level activity scores. The colour gradient represented the spectrum of activity scores from very low to very high

Applying the rationale and methodology described below and shown in Fig. 1, CYP2D6 phenotype activity scores varied between regions, but across the high burden *P. vivax* areas the proportion of potentially ineffective primaquine metabolizers was found greater than 25%. This was highest among East and Southeast Asian populations, where available data suggested that over a third of individuals were low or non-functional CYP2D6 metabolizers. In contrast, Melanesian populations are reported to have < 5% poor metabolizers.

## Primaquine ineligibility frequencies

Combining ineffective primaquine metabolism with the population excluded from therapy on safety grounds, an estimated 38.8% of the population living at risk of *P. vivax* infection is unable to receive safe and effective primaquine therapy. This corresponds to more than one in three potential *P. vivax* infections being untreatable with the only currently licensed radical cure drug. Populations in Africa combine both high prevalence of G6PD deficient mutations and poor CYP2D6 metabolizers so have the lowest regional proportions of primaquine-eligible hosts, but are at relatively much lower risk of *P. vivax* infection than populations elsewhere. High *P. vivax* burden regions such as the Western Pacific, Southeast Asia and the Americas have 59.5, 63.3 and 68.6% of their populations estimated to be eligible for safe and effective primaquine therapy, respectively. In three countries of the Mekong region, less than 50% of the population was found able to receive for safe and effective access to primaquine.

Uncertainty around these estimates was not quantified in this exploratory analysis, but would be an important next step. A robust model framework would allow the uncertainty around each exclusion step to be propagated through the exclusion stages to represent overall uncertainty in the final estimate and help to identify the most significant gaps in the evidence-base underlying this analysis. The use of predictive modelling could also help account for variability in the panel of CYP2D6 SNPs screened for between studies, with gaps accounted for through cross-learning between survey findings.

## More work on the primaquine ineligibility problem

The coarse-resolution analysis discussed here bears important limitations which need to be addressed through further development. G6PD deficiency is considered as a single universally preclusive condition, without accounting for the possibility of a protective effect of G6PD deficiency against infection, or of alternative treatment regimens which may be safely administered to *P. vivax* patients with certain levels of G6PD deficiency, such as 8 weekly dosing or a variable daily-dosing regimen [17]. The G6PD population estimates used came from a mapping analysis published in 2012 [3], since when there has been increased commitment to *P. vivax* elimination and the need to increase access to safe radical cure treatment [18, 19]. This momentum has led to a rise in the coverage of G6PD population surveys [20], which need to be integrated into a revised map and population estimates.

The importance of CYP2D6 metabolism in the context of primaquine is only recently being realized [11], despite being well known as necessary to the metabolism of 20–25% of drugs in clinical use [21]. Data on the impact of the gene’s extensive diversity on primaquine metabolism specifically is, therefore, limited (J. Kevin Baird, personal communication, 2017), forcing this present analysis to make assumptions that should be revisited as future studies offer greater insight into CYP2D6 metabolism of primaquine. For example, although the phenotypic categories described here provide useful indicators of CYP2D6 metabolism, heterogeneity in genotype–phenotype correspondence has been reported both within and between population groups: the same genotype may differ in phenotype between regions and ethnic groups [16, 22]. Further, the translation of genotypes into primaquine metabolism capacity is based here on a single threshold of an activity score of > 1.0 being considered sufficient to ensure *P. vivax* radical cure. However this is based on a single study of a single population group in Indonesia, and even within that sample, there is variation in relapse incidence among individuals of similar activity scores (J. Kevin Baird, personal communication, 2017). The fixed threshold of activity scores ≤ 1 representing inadequate metabolism to treat *P. vivax* is, therefore, variable and may be more permeable than considered here; an undefined proportion of low/intermediate CYP2D6 phenotypes may metabolize sufficient primaquine for therapeutic success. Furthermore, CYP2D6 activity scores are informed by metabolic rates with a range of substrates despite metabolic activity being observed to vary between compounds. Extensive characterization of primaquine metabolism is needed to develop a more intricate model of treatment failure risks in terms of CYP2D6 metabolism. In addition, the populations sampled in the database used to generate regional CYP2D6 characteristics were not always nationally representative and at times focused on minority population sub-groups. Other more nuanced technical issues^2^ must also be considered in the exceptionally complex problem of primaquine ineligibility.

## Conclusions

The complex haemolytic toxicity problem of primaquine in G6PD deficient patients puts that drug out of the reach of approximately 14% of people living at risk of infection by *P. vivax* (assuming an equal risk of infection across the population, see Footnotes 1, 2). Impaired CYP2D6 alleles tentatively linked to high risk of primaquine treatment failure occur in over 20% of those populations. If one-third or more of people infected by *P. vivax* cannot safely receive effective primaquine therapy against latent hypnozoites, alternative chemotherapeutic or chemoprophylactic approaches to preventing the multiple recurrent hypnozoite-borne clinical attacks of vivax malaria must be conceived, optimized, and validated. Moreover, achieving more robust estimates of these population groups will directly inform the strategy and practice of safely and effectively attacking the hypnozoite reservoir in human communities.

## Abbreviations

G6PD: glucose-6-phosphate dehydrogenase
CYP2D6: cytochrome P-450 isotype 2D6

## Footnotes

1 We reviewed published evidence of CYP2D6 metabolic activity estimates and assigned an activity score to each allele in the database of Sistonen et al. [15]. Genotype frequencies for each population were then derived according to the Hardy–Weinberg laws of inheritance, with resulting phenotypes allocated activity scores which were the sum of the component alleles’ scores (see Table 1). Resulting phenotypic activity scores ≤ 1.0 were deemed to be at high risk of primaquine therapeutic failure, as per the outcomes of the clinical trial by Baird et al. (J. Kevin Baird, personal communication, 2017). Each population group was thus assigned a proportion of impaired CYP2D6 alleles that would be at greatly elevated risk of primaquine treatment failure. An important limitation of the population surveys in the CYP2D6 database employed here [15] was the surveys’ focus on specific ethnic groups who are not necessarily representative samples of the wider population from that country. To account for this and for variability in the sample sizes across the dataset as well as the gaps in coverage across most countries, the present analysis used regional mean values for CYP2D6 population characteristics.

2 The regional-level CYP2D6 dataset used in this analysis masks significant heterogeneity between populations [15]. Increasing the resolution of the analysis to account for population-specific characteristics of CYP2D6 diversity is necessary. However, the *CYP2D6* gene has also been found to be highly polymorphic within populations, and lacking in continental structure [23]. The near-decade since publication of the Sistonen database has seen an increased research focus on CYP2D6. A Pubmed summary of publications including terms “CYP2D6” and “population” in August 2017 indicates a doubling of published articles since the Sistonen database was compiled in 2007. An updated literature review would therefore allow the feasibility of a sub-regional analysis to be assessed, something which would add significant resolution over the present coarse mapping of CYP2D6 activity scores. The selected focus of most surveys on specific ethnic groups, who in many cases may be minority subsets of overall populations, further limits the representativeness of the currently applied CYP2D6 dataset. For example, the population samples included from the Americas are all Amerindian communities, thus cannot be considered widely representative. To develop a representative database and map of CYP2D6 primaquine-metabolism capacity, inclusion criteria similar to those applied to the G6PD dataset used in this analysis are needed. Another nuance to consider is that the denominator applied here is the overall population living at any risk of *P. vivax* infection. This does not account for the relative risk of infection and consequent demand for primaquine. G6PD deficiency has been found to afford a protective effect against malaria, though the specific association of G6PD variants with *P. vivax* infection in particular remains poorly defined [4, 24]. A protective effect, however, would reduce the demand for primaquine by this group of ineligible patients, and mean that a greater proportion of *P. vivax* patients than indicated here are in fact eligible for treatment. Conversely, pregnant women are a well-known risk group for malaria, including *P. vivax* [25]. This group may therefore be disproportionately frequently represented among *P. vivax* patients requiring primaquine. Adjusting the proportion of ineligibles to the number of *P. vivax* cases, rather than the denominator of overall population size, would help increase the specificity of estimates of the proportion of cases ineligible for primaquine therapy.
